# Multi-omics integration reveals a six-malignant cell maker gene signature for predicting prognosis in high-risk neuroblastoma

**DOI:** 10.3389/fninf.2022.1034793

**Published:** 2022-11-10

**Authors:** Zijun Yan, Qiming Liu, Ziyang Cao, Jinxia Wang, Hongyang Zhang, Jiangbin Liu, Lin Zou

**Affiliations:** ^1^Clinical Research Unit, Shanghai Children’s Hospital, Shanghai Jiao Tong University School of Medicine, Shanghai, China; ^2^Institute of Pediatric Infection, Immunity, and Critical Care Medicine, Shanghai Jiao Tong University School of Medicine, Shanghai, China; ^3^State Key Laboratory of Genetic Engineering and Collaborative Innovation Center for Genetics and Development, Department of Computational Biology, School of Life Sciences, Fudan University, Shanghai, China; ^4^Department of General Surgery, Shanghai Children’s Hospital, Shanghai Jiao Tong University School of Medicine, Shanghai, China; ^5^Center for Clinical Molecular Laboratory Medicine of Children’s Hospital of Chongqing Medical University, Chongqing, China

**Keywords:** multi-omics integration, high-risk neuroblastoma, malignant cell maker gene, single-cell, prognostic model

## Abstract

**Background:**

Neuroblastoma is the most common extracranial solid tumor of childhood, arising from the sympathetic nervous system. High-risk neuroblastoma (HRNB) remains a major therapeutic challenge with low survival rates despite the intensification of therapy. This study aimed to develop a malignant-cell marker gene signature (MMGS) that might serve as a prognostic indicator in HRNB patients.

**Methods:**

Multi-omics datasets, including mRNA expression (single-cell and bulk), DNA methylation, and clinical information of HRNB patients, were used to identify prognostic malignant cell marker genes. MMGS was established by univariate Cox analysis, LASSO, and stepwise multivariable Cox regression analysis. Kaplan–Meier (KM) curve and time-dependent receiver operating characteristic curve (tROC) were used to evaluate the prognostic value and performance of MMGS, respectively. MMGS further verified its reliability and accuracy in the independent validation set. Finally, the characteristics of functional enrichment, tumor immune features, and inflammatory activity between different MMGS risk groups were also investigated.

**Results:**

We constructed a prognostic model consisting of six malignant cell maker genes (MAPT, C1QTNF4, MEG3, NPW, RAMP1, and CDT1), which stratified patients into ultra-high-risk (UHR) and common-high-risk (CHR) group. Patients in the UHR group had significantly worse overall survival (OS) than those in the CHR group. MMGS was verified as an independent predictor for the OS of HRNB patients. The area under the curve (AUC) values of MMGS at 1-, 3-, and 5-year were 0.78, 0.693, and 0.618, respectively. Notably, functional enrichment, tumor immune features, and inflammatory activity analyses preliminarily indicated that the poor prognosis in the UHR group might result from the dysregulation of the metabolic process and immunosuppressive microenvironment.

**Conclusion:**

This study established a novel six-malignant cell maker gene prognostic model that can be used to predict the prognosis of HRNB patients, which may provide new insight for the treatment and personalized monitoring of HRNB patients.

## Introduction

Neuroblastoma (NB) is one of the most common malignant solid tumors of childhood, accounting for 15% of childhood cancer-related deaths (Maris et al., 2007). NB is characterized by extensive heterogeneous clinical phenotype, ranging from spontaneous regression to metastatic disease with poor prognosis (George et al., 2020). The current risk classification system uses clinical and biological variables, such as age, stage, and MYCN oncogene amplification, to stratify NB patients into three risk groups (low-, intermediate-, and high-risk) (Cohn et al., 2009). The survival probability for low-risk patients exceeds 90%, however, it remains below 50% (Ladenstein et al., 2017; Amoroso et al., 2018) in high-risk neuroblastoma (HRNB) patients despite intensive and multi-modal therapy. In addition, patients with an inherently good prognosis but classified into the high-risk group under the current stratification system will undergo toxic treatment, which exposes them to an unnecessary risk of potential long-term side effects (Vermeulen et al., 2009). Therefore, it is imperative to develop more precise biomarkers for HRNB patients to avoid under- or over-treatment and to discriminate who will benefit from new experimental therapy.

The advent of high-throughput sequencing technologies has led to increased efforts to identify molecular prognostic markers in NB from various omics (De Preter et al., 2011; Decock et al., 2012; Garcia et al., 2012; Valentijn et al., 2012; He et al., 2020; Wang et al., 2020), partly with the desire to refine existing risk stratification further. Previous studies have only used dysregulated genes (Chen et al., 2016; Wei et al., 2018) or genomic alterations (Bilke et al., 2008; Stigliani et al., 2012; Depuydt et al., 2018; Fernandez-Blanco et al., 2021) to predict HRNB survival, rarely through multi-omics integration. Additionally, these studies largely used bulk sequencing technologies, which were restricted to mixed cell populations. Therefore, despite successfully identifying many prognostic genes, these studies heavily ignored tumor heterogeneity. NB is a type of tumor intimately related to the early development and differentiation of neuroendocrine (NE) cells (Nunes-Xavier et al., 2021). The intrinsic and extrinsic features of malignant NE cells can be precisely characterized using single-cell RNA sequencing (scRNA-seq) technology, which enables gene profiling and discovery of oncogenic cellular populations and associated marker genes at single-cell resolution (Tang et al., 2009; Patel et al., 2014). Thus, the lack of prognostic stratification for HRNB based on scRNA-seq data and multi-omics integration analysis prompted us to conduct this study.

In this study, we first utilized scRNA-seq data from HRNB patients to identify marker genes of malignant cells. Subsequently, a malignant-cell marker gene signature (MMGS) was constructed to predict the prognosis of HRNB patients based on multi-omics integration analysis. Furthermore, the prognostic value and performance of MMGS were validated in an independent cohort. Finally, the relationships between MMGS and tumor immune/inflammatory features were investigated.

## Materials and methods

### Data acquisition and preprocessing

ScRNA-seq data of 11 HRNB samples and cell types annotation file were downloaded from GSE137804 (Dong et al., 2020) and were exploited to identify the marker genes of malignant cell. Meta-program gene sets (1 and 6) highly expressed in HRNB and strongly associated with a poor prognosis were downloaded from Dong et al. (2020). Bulk DNA methylation and bulk mRNA expression datasets of 56 HRNB patients were downloaded from GSE73515 and GSE73517, respectively (Henrich et al., 2016). Sequencing Quality Control (SEQC) (Zhang et al., 2015) and Therapeutically Applicable Research to Generate Effective Treatments (TARGET) (Pugh et al., 2013) HRNB cohorts with bulk RNA-seq profiles and clinical information were downloaded from GSE49711 and TARGET datasets,^1^ respectively. Gene mutation profiles of HRNB samples were downloaded from the TARGET project.^2^

The “Seurat” package (Butler et al., 2018) was employed to analyze and visualize scRNA-seq data. The “FindAllMarkers” function was used to identify the marker genes of malignant cells. Adjusted *p*-value < 0.05 and log2(fold change) > 0.25 were used as the cutoff threshold values to identify marker genes. The “RunUMAP” function was used to embed the cells in a two-dimensional map. For bulk RNA expression data, quantile algorithm from “limma” package (Ritchie et al., 2015) was used to perform normalization. For bulk DNA methylation data, subset quantile normalization was applied according to Touleimat and Tost (2012). We removed the sites missing in more than 30% of the samples and filled the missing values using the k-Nearest Neighbor algorithm. Pearson’s correlation coefficient of each gene corresponding to bulk mRNA expression and bulk DNA methylation profile was calculated. Genes with an absolute correlation coefficient greater than 0.4 and *p*-value < 0.001 were identified as methylation correlated (METcor) genes. The overlap between malignant cell marker genes and METcor genes was named MK-METcor genes and used for further study.

### Construction and validation of malignant cell marker genes prognostic model

A univariate Cox regression analysis was conducted to assess the prognostic value of MK-METcor genes for overall survival (OS) in SEQC HRNB cohort. *P*-value < 0.05 was used as the cutoff threshold value to identify as a prognostic gene. Next, least absolute shrinkage and selection operator (LASSO) Cox regression (Tibshirani, 1997) analysis was conducted to evaluate the prognostic genes using the “glmnet” package. Finally, a stepwise multivariate Cox regression analysis was used to optimize the prognostic signatures. A linear combination of the risk coefficient and mRNA expression of the genes was used to construct a risk model. Based on the median risk score, HRNB patients were divided into two risk groups: the ultra-high-risk group (UHR group) and the common-high-risk group (CHR group). The Kaplan–Meier (KM) method and the log-rank test were used to calculate the statistical significance of the differences in survival using the “survminer” package. The area under the curve (AUC) of the time-dependent receiver operating characteristic (tROC) curve was calculated to validate the prognostic power of MMGS using the “survivalROC” package (Heagerty and Zheng, 2005). The prognostic ability of MMGS was validated in an independent TARGET HRNB cohort. Clinical model was constructed based on canonical clinical and molecular variables (including age, sex, and MYCN status) by multivariable cox regression.

### Differentially expressed gene identification and enrichment analysis

The “limma” package (Ritchie et al., 2015) was used to identify DEGs between UHR and CHR groups. Adjusted *p*-value < 0.05 and fold change (FC) > 1 were used as the cutoff threshold values to identify differentially expressed genes (DEGs). Compared with the CHR group, DEGs that were overexpressed in the UHR group were considered “overexpressed DEGs” and those that were underexpressed in the UHR group were considered “underexpressed DEGs.”

Gene ontology (GO) and Kyoto Encyclopedia of Genes and Genomes (KEGG) pathway enrichment analyses were conducted using the “enrichGO” and “enrichKEGG” functions in the “clusterProfiler” (Yu et al., 2012) package, respectively. The enriched terms were filtered with the *p*-value adjusted by the Benjamini-Hochberg method (adj*P*-value) < 0.05.

Gene Set Enrichment Analysis (GSEA) was performed using the “gseKEGG” and “GSEA” functions in the “clusterProfiler” package (Yu et al., 2012) with gene sets involved in KEGG pathways and hallmarks, respectively. Hallmark gene sets were download from Molecular Signatures Database (MsigDB) (h.all.v6.2.symbols.gmt) (Liberzon et al., 2015). False discovery rate (FDR) *q*-value < 0.05 and | NES (net enrichment score)| > 1 were set as the significant thresholds.

### Tumor immune and inflammatory features analyses

The ESTIMATE algorithm was employed to assess infiltration levels of the immune and stromal cells by the “estimate” package (Yoshihara et al., 2013). The CIBERSORT algorithm (Newman et al., 2019) was used to dissect the proportion of 22 immune cell types infiltration.

Seven metagene clusters (HCK, Interferon, MHC-I, MHC-II, IgG, LCK, and STAT1) were collected from Rody et al. (2009) that have been widely applied to evaluate the tumor inflammatory activity. We conducted a single-sample GSEA (ssGSEA) analysis to obtain an inflammatory activity score by the “GSVA” package (Hanzelmann et al., 2013).

### Statistical analysis

All analyses were conducted with R version 4.0.0^3^ and its appropriate packages. Univariate and multivariate Cox regression analyses were performed to assess the prognostic value of MMGS and various clinical variables. Wilcoxon test or Kruskal–Wallis test was used to analyze differences between different groups. The chi-square test was conducted to investigate the association between MMGS risk groups, MYCN status, and OS status. The “maftools” package (Mayakonda et al., 2018) was used to visualize the genomic mutation landscape. Heatmap plot was visualized using the “pheatmap” package. *P*-value < 0.05 was set as a significant threshold.

## Results

### Identification of malignant cell marker genes expression profiles

**FIGURE 1:**
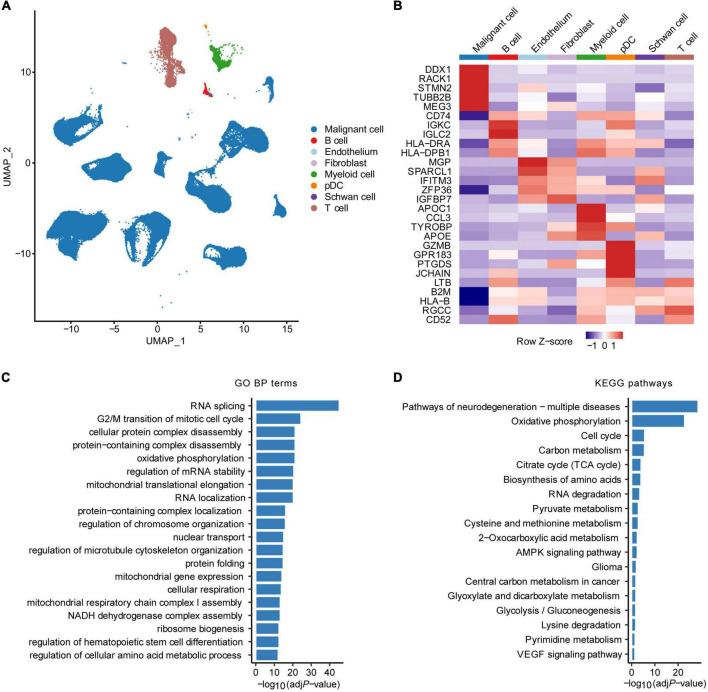
Identification of malignant cell marker genes by scRNA-seq analysis. **(A)** UMAP plot colored by various cell types. **(B)** Heatmap showing the top five marker genes of each cell type. GO biological process (BP) terms **(C)** and KEGG pathways **(D)** enrichment analysis of malignant cell marker genes of HRNB. pDC, plasmacytoid dendritic cell.

The overall flowchart of this study was shown in Supplementary Figure 1. First, we obtained scRNA-seq data of 160,910 cells from 11 HRNB patients, including 96,843 malignant NE cells (Dong et al., 2020; Figure 1A). A total of 3,404 malignant cell marker genes were identified (Figure 1B). We observed that malignant cells highly expressed signature genes of chromaffin cells (such as STMN2, TUBB2B, and MEG3), presenting consistent results reported by Dong et al. (2020) that most cancer cells showed a strong chromaffin-cell-like feature. We found that the malignant cell marker genes were enriched in pathways associated with cell cycle and metabolic features, such as oxidative phosphorylation, carbon metabolism, citrate cycle (TCA cycle), regulation of the cellular amino acid metabolic process, and pyruvate metabolism (Figures 1C,D).

### Construction of malignant-cell marker gene signature prognostic model

Based on the correlation between bulk mRNA expression and bulk DNA methylation, a total of 829 METCor genes were obtained (Supplementary Figure 2A), mainly located on the CpG island (Supplementary Figure 2B). The 168 MK-METcor genes, overlapping between malignant cell marker genes and METcor genes, were used for subsequent analysis (Supplementary Figure 2C).

**FIGURE 2:**
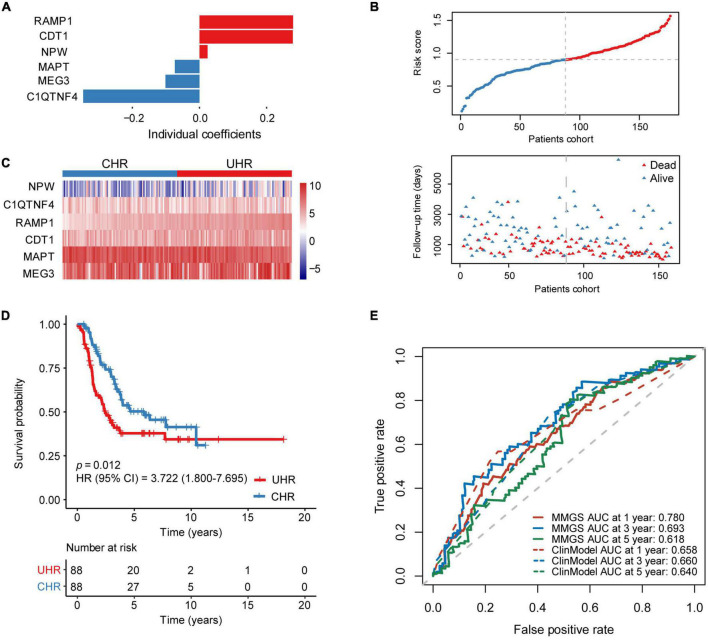
Establishment of MMGS in the SEQC HRNB cohort. **(A)** The coefficients of the identified six malignant cell marker genes. **(B)** The distribution of risk score and survival status. **(C)** The expression characteristics of the identified six malignant cell marker genes. **(D)** KM curves of OS between the UHR and CHR groups. **(E)** ROC curves of MMGS model (solid lines) and clinical model (dot lines) to predict the 1-, 3-, and 5-year OS. ClinModel, clinical model; HR, hazard ratio; CI, confidence interval.

To construct a prognostic model based on the 168 MK-METcor genes, we first used 176 HRNB patients from the SEQC dataset as the training set to conduct a univariate Cox regression analysis. We identified 35 MK-METcor genes with prognostic value. Subsequently, LASSO Cox regression analysis was used to shrink the variants to 16 genes (Supplementary Figures 3A,B). Finally, stepwise multivariate Cox regression analysis was performed to optimize genes to include only the six most predictive genes. Therefore, MMGS risk score = (0.279 × RAMP1 expression) + (0.278 × CDT1 expression) + (0.024 × NPW expression) + (-0.073 × MAPT expression) + (-0.101 × MEG3 expression) + (-0.347 × C1QTNF4 expression) (Figure 2A). RAMP1, CDT1, NPW, and MAPT showed significant expression differences between CHR and UHR groups, while MEG3 and C1QTNF4 didn’t show expression differences between UHR and CHR groups (Supplementary Figure 3C). The relative expression of these six signature genes in various cell types showed their tumor cell-specific expression (Supplementary Figure 3D). Except for MAPT, the other genes showed a negative correlation between mRNA expression and DNA methylation (Supplementary Figure 3E). By ranking the risk score from low to high, the median risk score (0.901) was used to divide patients into CHR (*n* = 88) and UHR (*n* = 88) groups (Figure 2B). The expression profiles of six signature genes in the training set were shown in Figure 2C. KM analysis demonstrated that UHR patients had a significantly shorter OS than CHR patients (*p* = 0.012, HR: 3.722 [95% CI: 1.800–7.695]) (Figure 2D). A tROC analysis showed that the AUC values for the 1-, 3-, and 5-year OS of MMGS model were 0.780, 0.693, and 0.618, respectively, which were higher than those of the clinical model (except AUC for 5-year OS) (Figure 2E).

### Validation of the prognostic value of malignant-cell marker gene signature

**FIGURE 3:**
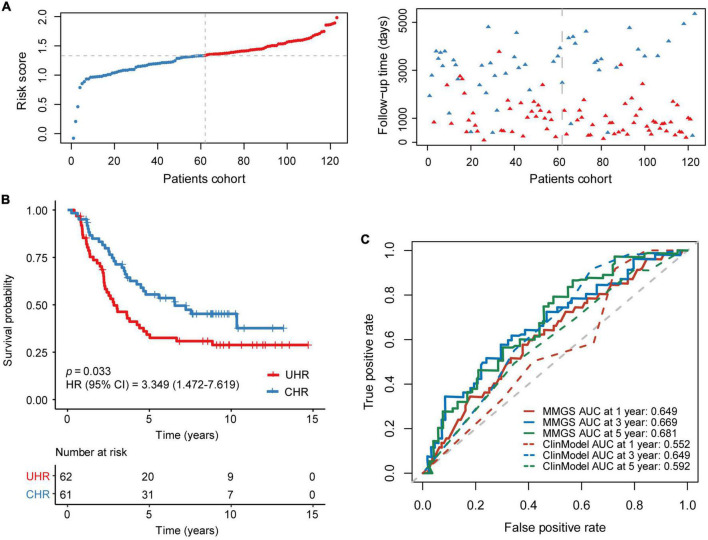
Validation of MMGS in the TARGET HRNB cohort. **(A)** The distribution of risk score and survival status. **(B)** KM curves of OS between the UHR and CHR groups. **(C)** ROC curves of MMGS model (solid lines) and clinical model (dot lines) to predict the 1-, 3-, and 5-year OS. ClinModel, clinical model; HR, hazard ratio; CI, confidence interval.

In an independent cohort including 123 HRNB patients from the TARGET project, a risk score was calculated for each patient. Taking the median risk score as the cutoff value, patients were classified into UHR (*n* = 61) and CHR (*n* = 62) groups (Figure 3A). KM analysis also showed that the UHR group had an inferior prognosis than the CHR group (Figure 3B, *p* = 0.033, HR: 3.557, [95% CI: 1.856–6.818]). A tROC analysis showed that the AUC values for 1-, 3-, and 5-year OS of MMGS model were 0.649, 0.669, and 0.681, respectively, which were higher than those of the clinical model (0.552, 0.649, and 0.592, respectively) (Figure 3C).

To further validate the superiority of MMGS, we compared MMGS model to the model constructed without DNA methylation information (not including METcor gene set) (Supplementary Figure 4A). This model consisted of eight signature genes (Supplementary Figure 4B). UHR patients had a significantly shorter OS than CHR patients (*p* = 0.0011 and 0.042, respectively) in both training and validation sets (Supplementary Figures 4C,D). The AUC values for the 3- and 5-year OS (0.727, 0.675, respectively) of this model were slightly higher than those of MMGS in the training set (Supplementary Figure 4E). However, in the validation set, the AUC values for 1-, 3- and 5-year OS of this model (0.626, 0.632, and 0.562, respectively) were lower than those of the MMGS model (Supplementary Figure 4F).

Additionally, Dong et al. (2020) identified two meta-program gene sets (1 and 6), which were highly expressed in HRNB and strongly associated with a poor prognosis. Then, we compared MMGS model to the model starting with these two meta-program gene sets and with (Supplementary Figure 5) or without DNA methylation information (Supplementary Figure 6). The model constructed with meta-program gene sets and METcor gene set consisted of three signature genes (Supplementary Figures 5A,B). UHR patients had a significantly shorter OS than CHR patients (*p* = 0.0074) in the training set (Supplementary Figure 5C) and had a nearly significantly shorter OS than CHR patients (*p* = 0.053) in the validation set (Supplementary Figure 5D). The AUC values for the 3- and 5-year OS (0.72, 0.646, respectively) of this model were slightly higher than those of the MMGS model in the training set (Supplementary Figure 5E). However, in the validation set, the AUC values for 1-, 3- and 5-year OS of this model (0.633, 0.592, and 0.564, respectively) was all lower than those of the MMGS model (Supplementary Figure 5F). The model construction using metaprograms genes without DNA methylation information consisted of eight signature genes (Supplementary Figures 6A,B). UHR patients had a significantly shorter OS than CHR patients (*p* = 0.00053) in the training set (Supplementary Figure 6C). However, there was no significant difference in OS between UHR and CHR patients (*p* = 0.81) in the validation set (Supplementary Figure 6D). The AUC values for the 3- and 5-year OS (0.7, 0.706, respectively) of this model were slightly higher than those of MMGS model in the training set (Supplementary Figure 6E). However, in the validation set, the AUC values for 1-, 3- and 5-year OS of this model (0.546, 0.572, and 0.548, respectively) were all lower than those of MMGS model (Supplementary Figure 6F). These results indicated that construction of the model starting with meta-program gene sets had slight better performance in predicting 3- and 5-year OS in the training set. However, it’s performance in the validation set was lower than the MMGS model.

To further investigate whether MMGS model can be used to independently predict the survival of HRNB patients, we conducted univariate and multivariate Cox regression analyses using the risk score, clinical variables, and molecular features. As expected, multivariate Cox regression analysis demonstrated that MMGS model was an independent prognostic factor in the SEQC (Table 1, *p* = 0.039, HR: 1.585, [95% CI: 1.023–2.457]) and TARGET (Table 2, *p* = 0.008, HR: 1.910, [95% CI: 1.188–3.070]) cohorts.

**TABLE 1:** Univariate and multivariate analyses of MMGS model in the SEQC HRNB cohort.

	Univariate analysis		Multivariate analysis	
		
Variables	HR (95% CI)	*P*-value	HR (95% CI)	*P*-value
MMGS (UHR vs. CHR)	1.69 (1.119–2.553)	0.012	1.585 (1.023–2.457)	**0.039**
Age (y) (> 5 vs. =5)	1.333 (0.810–2.192)	0.257	1.546 (0.926–2.583)	0.096
Sex (Male vs. female)	0.571 (0.377–0.864)	0.007	0.656 (0.426–1.010)	0.055
MYCN (amp vs. non-amp)	1.708 (1.125–2.592)	0.011	1.466 (0.925–2.323)	0.104

HR, hazard ratio; CI, confidence interval; y, year.

*P*-values less than 0.05 in multivariate analysis were highlighted in bold.

**TABLE 2:** Univariate and multivariate analyses of MMGS model in the TARGET HRNB cohort.

	Univariate analysis		Multivariate analysis	
		
Variables	HR (95% CI)	*P*-value	HR (95% CI)	*P*-value
MMGS (UHR vs. CHR)	1.729 (1.090–2.744)	0.019	1.910 (1.188–3.070)	**0.008**
Age (y) (> 5 vs. = 5)	0.572 (0.329–0.997)	0.046	0.541 (0.309–0.947)	**0.031**
Sex (Male vs. female)	1.118 (0.706–1.772)	0.634	1.136 (0.713–1.810)	0.591
MYCN (amp vs. non-amp)	0.977 (0.568–1.682)	0.934	0.790 (0.453–1.376)	0.405

HR, hazard ratio; CI, confidence interval; y, year.

*P*-values less than 0.05 in multivariate analysis were highlighted in bold.

### The correlation of malignant-cell marker gene signature risk score with clinical variables

**FIGURE 4:**
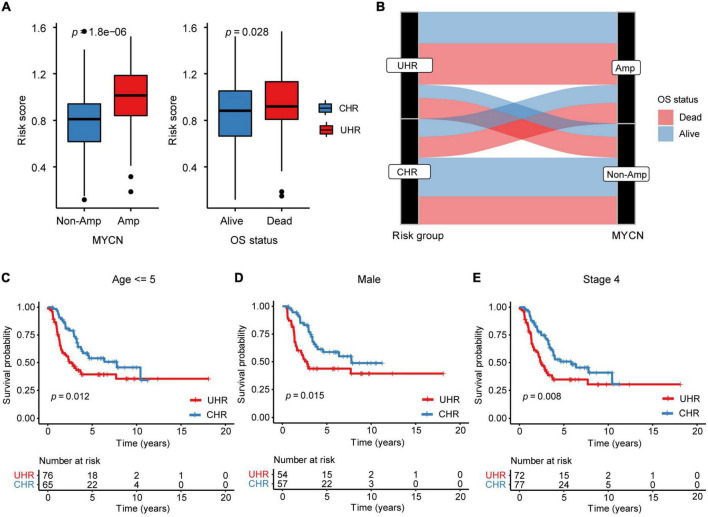
The relationship between MMGS risk score and clinical variables in the SEQC cohort. **(A)** The relationship between MMGS risk score and MYCN status, OS status. **(B)** Sankey diagram showing the association between MMGS risk score, MYCN status, and OS status. KM curves of OS between UHR and CHR patients with age less than 5 years old **(C)**, male **(D)**, and stage 4 **(E)**, respectively. Amp, amplified.

To examine the correlation between MMGS risk score and clinical features, we investigated MMGS risk score distribution in patients from the SEQC cohort. We observed that the risk score was lower in the non-MYCN-amplified and alive subgroups whereas higher in MYCN-amplified and dead subgroups (Figure 4A and Supplementary Figure 7A). The sankey diagram was employed to visualize the association between MMGS risk group, MYCN status, and OS status (Figure 4B). We found that patients in the HRNB group had a higher proportion of MYCN amplification with the dead outcome (chi-square test, *p* = 0.003). Moreover, we found that significantly poorer survival probability was associated with high risk scores in HRNB patients with different clinical features, including age (=5 years), male, and stage 4 (Figures 4C–E and Supplementary Figure 7B). In addition, we also compared the difference in event-free survival (EFS) between UHR and CHR groups and observed almost the same results compared with results of overall survival (Supplementary Figures 7C,D).

### Functional enrichment analysis of malignant-cell marker gene signature risk groups

**FIGURE 5:**
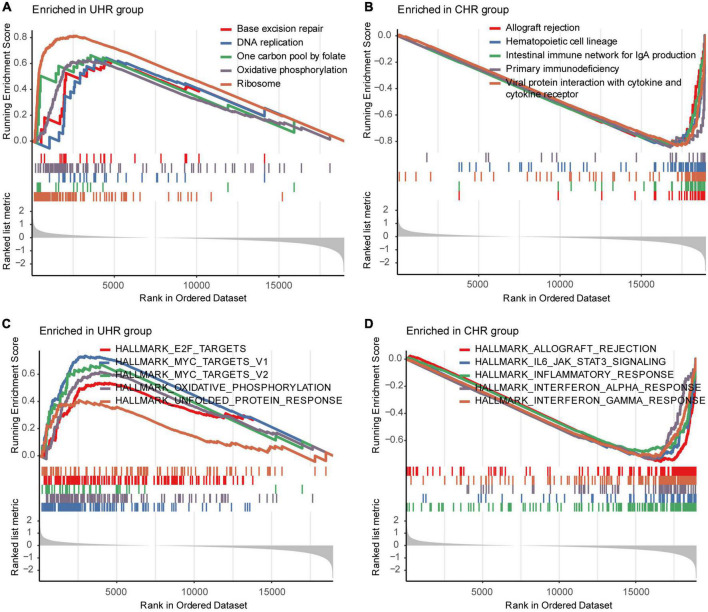
Representative pathways enriched in UHR and CHR groups from the SEQC cohort. The top five significantly enriched KEGG pathways in the UHR **(A)** and CHR **(B)** HRNB patients. The top five significantly enriched hallmarks in the UHR **(C)** and CHR **(D)** HRNB patients.

To explore transcriptomic differences between MMGS risk groups, we further investigated pathways and hallmarks enriched in UHR and CHR groups from the SEQC cohort. Differential expression analysis showed UHR group highly expressed metabolic-related genes, such as HTR1E and KCNH5, while CHR group highly expressed inflammatory-related genes, such as CNR2, TNFSF11, CCL21, and CCL19 (Supplementary Figure 8A). We found that the UHR group significantly enriched in the metabolic-related pathways such as oxidative phosphorylation, and MYC target hallmarks (Figures 5A,C), whereas the CHR group enriched in immune- and inflammatory-related pathways like IL6_JAK_STAT3 signaling, inflammatory response, and interferon alpha/gamma response (Figures 5B,D). Consistent results were also observed in the TARGET cohort (Supplementary Figures 8B–E).

### Malignant-cell marker gene signature risk score was associated with tumor immune and inflammatory features

**FIGURE 6:**
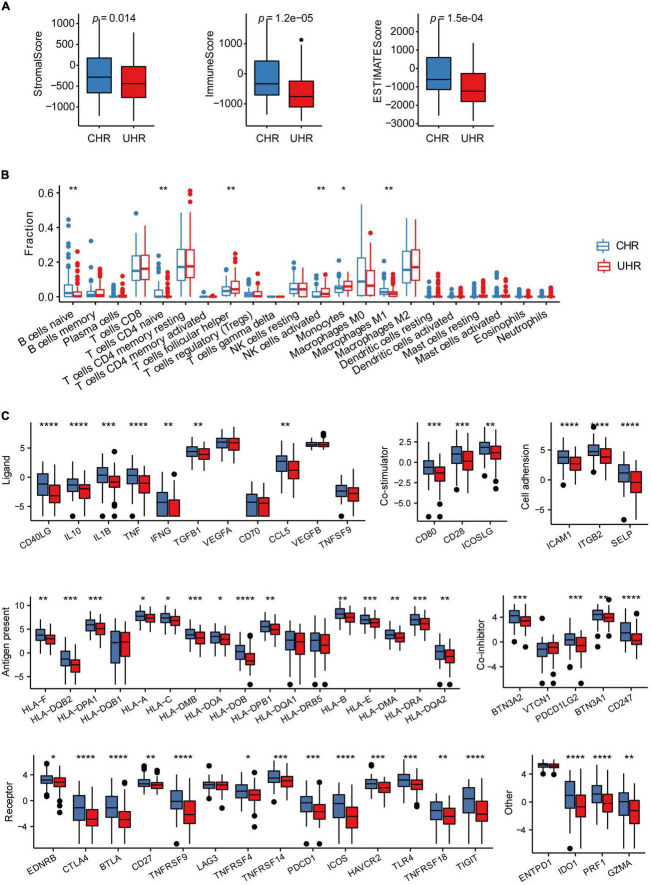
The association between MMGS and the immune cell infiltration in the SEQC cohort. **(A)** Differences among stromal score, immune score, and ESTIMATE score between UHR and CHR groups. **(B)** The comparison of infiltration levels of 22 immune cell types between UHR and CHR groups. **(C)** Immune checkpoints expression between UHR and CHR groups. *p < 0.05; **p < 0.01; ***p < 0.001; ****p < 0.0001; ns, no significance.

To assess the potential efficacy of immunotherapies in MMGS risk groups, we explored the relationship between MMGS and tumor immune features in the HRNB microenvironment in the SEQC cohort. Based on the ESTIMATE algorithm, we observed that UHR group had significantly lower immune, stromal, and ESTIMATE scores than CHR group (Figure 6A). Next, using the CIBERSORT algorithm, we found that UHR patients had a higher fraction of T cells follicular helper, NK cells activated, and monocytes but had a lower fraction of B cells naïve, T cells CD4 naïve, and macrophage M1 (Figure 6B). Previous studies have reported that B cells promoted immunotherapy response (Helmink et al., 2020) and macrophages functioned as regulators of tumor immunity and immunotherapy (DeNardo and Ruffell, 2019). Characterization of immune cell infiltration levels might provide implications for the development of immunotherapy targets. Furthermore, we compared the expression levels of immune checkpoints and immune-activity-related genes between UHR and CHR groups. The results demonstrated that most of these genes had relatively lower expression in the UHR group (Figure 6C), suggesting that such tumors might be less responsive to immunotherapies.

**FIGURE 7:**
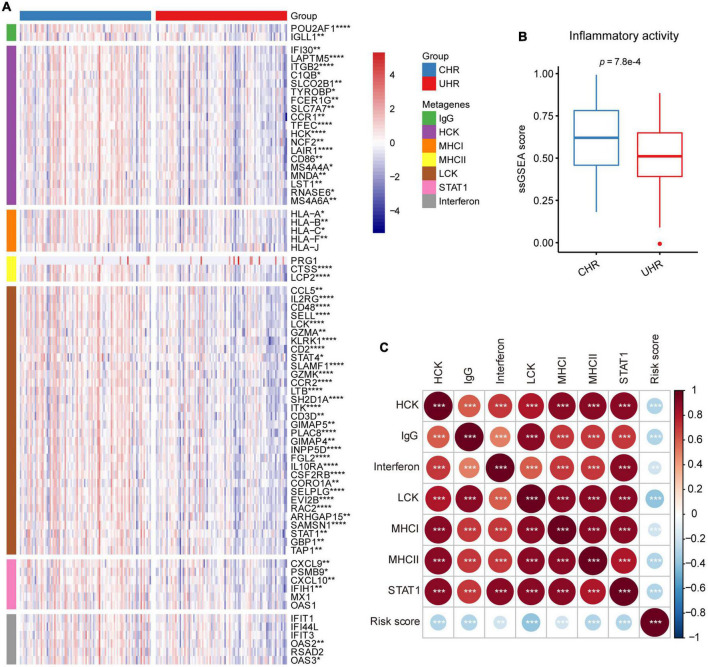
Relationship between MMGS and inflammatory metagenes in the SEQC cohort. **(A)** Heatmap showing the expression characteristics of seven clusters of metagenes. **(B)** The ssGSEA score of inflammatory activity in MMGS risk groups. **(C)** The correlation between MMGS risk scores and metagenes. *p < 0.05; **p < 0.01; ***p < 0.001.

To explore the relationship between MMGS risk scores and tumor inflammatory activities, we investigated the associations between MMGS risk scores and seven metagene clusters. These metagene clusters (HCK, Interferon, MHC-I, MHC-II, IgG, LCK, and STAT1) have previously been reported to represent various tumor inflammatory activities (Rody et al., 2009). We found that most of the metagenes were significantly lower expressed in the UHR group (Figure 7A). Next, we found that the UHR group had a lower inflammatory activity score than the CHR group based on ssGSEA analysis (Figure 7B). Moreover, we observed that MMGS risk score showed negative correlations with all clusters (Figure 7C). Similar results were also observed in the TARGET cohort (Supplementary Figures 9, 10).

These significant differences in immune and inflammatory features between the UHR and CHR groups inspired us to explore whether these features can improve the performance of MMGS model. Thus, we combined these features into MMGS to construct seven kinds of models. In the training set, AUC values for 1- and 5-year OS of these models were lower than those of MMGS model; AUC values for 3-year OS were close to that of MMGS model (Supplementary Figure 11A). In the validation set, AUC values for 1- and 5-year OS were close to those of MMGS model; AUC values for 3-year OS were lower than those of MMGS model (Supplementary Figure 11B). These results showed that these immune and inflammatory features didn’t effectively seem to improve the performance of MMGS model.

### Mutation landscape of malignant-cell marker gene signature risk groups

We further examined the mutation profiles of MMGS risk groups. ALK stood out that it’s the highest mutation frequency (Supplementary Figure 12A). It was proven that ALK gene mutation was a susceptibility factor and a crucial molecular driver of NB (Mosse et al., 2008). In addition, we found that patients with ALK mutation nearly correlated with poor survival compared to the wild-type (WT) patients in the UHR group (Supplementary Figure 12B, *p* = 0.06).

## Discussion

The majority of HRNB is resistant or refractory to the current intensive multimodality therapy, and the survival rates of these patients remain poor (Irwin et al., 2021). ScRNA-seq technology combined with multi-omics data could help to dissect tumor cell heterogeneity and identify potential prognostic biomarkers effectively. Therefore, we first screened HRNB scRNA-seq data to identify 3,404 malignant cell marker genes and found 35 prognostic MK-METcor genes based on multi-omics integration analysis. After that, we developed a six-malignant cell marker cell signature, termed MMGS, which could serve as a novel and independent indicator for the prognosis of HRNB patients. We found that the CHR group had higher levels of tumor-infiltrating immune cells and inflammatory activity. In addition, we discovered that the genes related to immune checkpoints had higher expression in the CHR group than in the UHR group, indicating that immunotherapy might be more appropriate for CHR patients.

Malignant cell marker genes included in our model are: MAPT, C1QTNF4, MEG3, NPW, RAMP1, and CDT1. MAPT is a gene encoding tau protein, which is closely related to maintaining the function of microtubules. It was reported that MAPT might affect tumorigenesis through dysregulation of cell cycle progression, cell mobility, or organelle organization (Papin and Paganetti, 2020). Zaman et al. (2018) reported that MAPT expression was a biomarker for an increased survival rate in pediatric NB. C1QTNF4 belongs to the C1q and TNF-related family. Previous studies suggested its roles in cancer-related inflammation (Li et al., 2011; Luo et al., 2016) and metabolism (Li et al., 2020; Sarver et al., 2020). MEG3 is the first lncRNA discovered to have tumor suppressor function (Beygo et al., 2015). MEG3 expression is controlled by epigenetics, and it is aberrantly CpG-methylated in various tumors (Modali et al., 2015). MEG3 was reported to be significantly downregulated in NB and negatively associated with the international neuroblastoma staging system (INSS) stage (Ye et al., 2020). Moreover, Novak et al. (2020) reported that MEG3 and EZH2 regulated each other through a negative feedback loop and jointly promoted NB progression, suggesting MEG3 may be a potential treatment target for NB. NPW is a gene encoding neuropeptide protein, which can enhance cortisol secretion from adrenal cells. RAMP1 is a chaperone to the amylin and calcitonin-gene-related peptide (CGRP) receptors. It has been reported that RAMP1 is expressed in various tissues, including the adrenal gland (Nagae et al., 2000; Cottrell et al., 2005). CDT1 plays a pivotal role in cell replication and cell cycle regulation (Yang et al., 2019). The destructive role of CDT1 has been demonstrated in tumorigenesis, progression, and chemoresistance in certain tumor types (Karakaidos et al., 2004; Bravou et al., 2005). These reports suggested that genes identified in our MMGS model might play pivotal roles in malignant cells’ biological behaviors and were potential targets for elucidating molecular mechanisms of HRNB.

In this study, MMGS model was proved to be an independent prognostic tool for HRNB tumors in both training and validation datasets. The excellent prognostic value and performance of MMGS motivated us to explore the potential molecular mechanism. We first conducted GSEA analysis, including KEGG pathway and hallmarks, in MMGS risk groups and observed that the UHR group was significantly enriched in metabolic-related pathways, whereas the CHR group was enriched in immune- and inflammatory-related pathways. Thus, the inferior prognosis of UHR group may be partly due to the dysregulation of the metabolic process, which plays a crucial role in tumor progression (Faubert et al., 2020). Additionally, tumor-infiltrating immune cells have been reported to affect tumor development and prognosis (Petitprez et al., 2020; Paijens et al., 2021). Then, we applied ESTIMATE and CIBERSORT algorithms to compare the levels of immune cell infiltration between UHR and CHR groups. The results demonstrated that the UHR group had a lower level of immune cell infiltration, suggesting that this group has a characteristic of “cold tumors” with reduced antitumor activity (Bonaventura et al., 2019). Low levels of tumor-infiltrating immune cells can facilitate tumor cells to evade immune surveillance and promote malignant progression (Song et al., 2022), which may partially be responsible for the inferior prognosis in UHR patients. Furthermore, we found that most of immune checkpoint genes had relatively lower expression in the UHR group. Besides, we observed that most of the inflammatory-related metagenes also had lower expression in the UHR group. Additionally, MMGS risk score showed negative correlations with all metagenes clusters. These results indicated that UHR patients might be in a more immunosuppressive microenvironment, which may partly account for the decreased OS of UHR tumors. Based on the above findings, we indicated that the underlying mechanism for the powerful prognostic performance of MMGS may be due to the dysregulation of the metabolic process and immunosuppressive microenvironment.

Nevertheless, our study still has some limitations. Our prognostic model was constructed and validated based on retrospective cohorts. A large local cohort is need to further validate our model. Additionally, our study did not analyze the interaction of malignant cell marker genes with microenvironmental cell marker genes. Taken together, although there were still some limitations, our study provided a novel and independent indicator for the prognosis of HRNB patients and illuminated potential molecular mechanisms for HRNB.

## Conclusion

In conclusion, a six-malignant-cell marker gene signature was established and validated to have an independent performance in predicting the prognosis of HRNB patients. It may serve as a prognostic signature to better predict the prognosis of HRNB patients and help clinicians to develop personalized treatment for HRNB patients.

## Data availability statement

The datasets presented in this study can be found in online repositories. The names of the repositories and accession numbers can be found in the article/Supplementary material.

## Author contributions

LZ and JL guided and supervised this work. ZY designed the work and participated in data collecting, data processing, program implementation, and manuscript writing. QL contributed to immune cell infiltration analysis. ZC, JW, and HZ contributed to the article polish. All authors provided critical advice for the final manuscript.

## Footnotes

1
https://ocg.cancer.gov/programs/target/data-matrix

2
https://portal.gdc.cancer.gov/

3
http://www.R-project.org
