# Supplementary material for: Multi-omics integration reveals a six-malignant cell maker gene signature for predicting prognosis in high-risk neuroblastoma
Source: Front Neuroinform. 2022 Nov 10;16:1034793. doi: 10.3389/fninf.2022.1034793 (PMC9687102; doi:10.3389/fninf.2022.1034793)
Supplement: Supplementary file 1 [file Data_Sheet_1.pdf]

Supplementary Material

1 Supplementary Figures

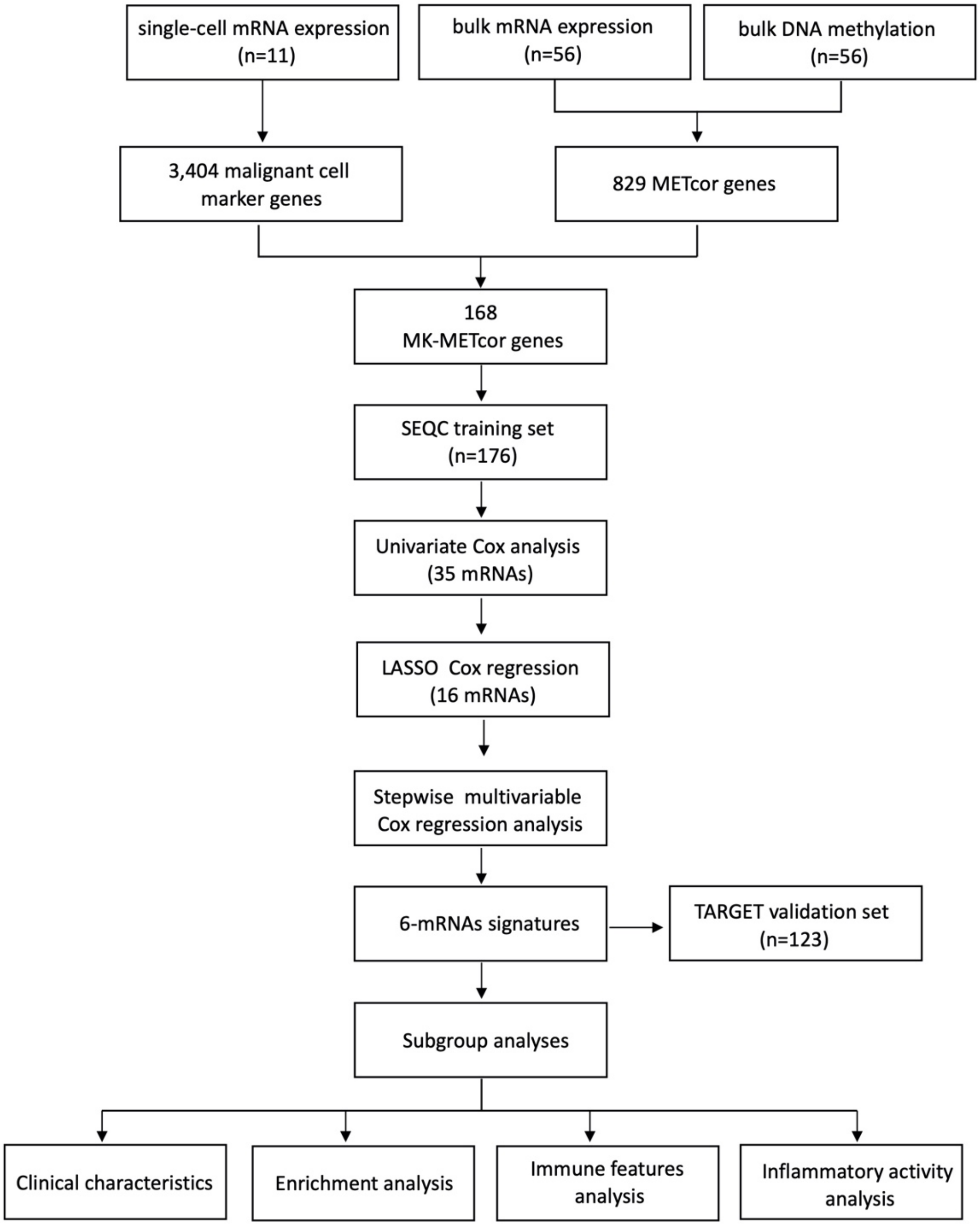

Supplementary Figure 1. Overall flowchart of this study. METcor: methylation correlated; MK-METcor: marker and methylation correlated; LASSO, least absolute shrinkage, and selection operator.

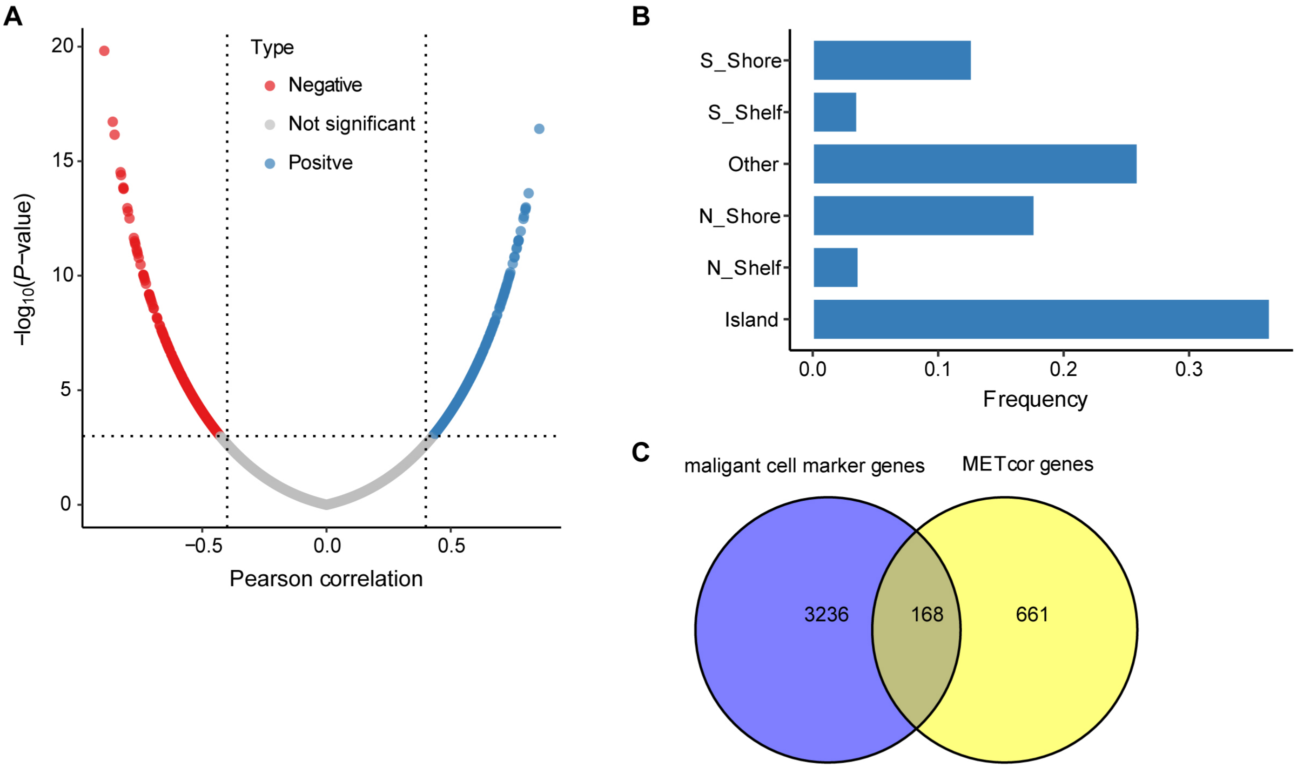

Supplementary Figure 2. The distribution of METcor genes. **(A)** The correlation between mRNA expression and DNA methylation. **(B)** The type of methylation sites. **(C)** The overlap between malignant cell marker genes and METcor genes.

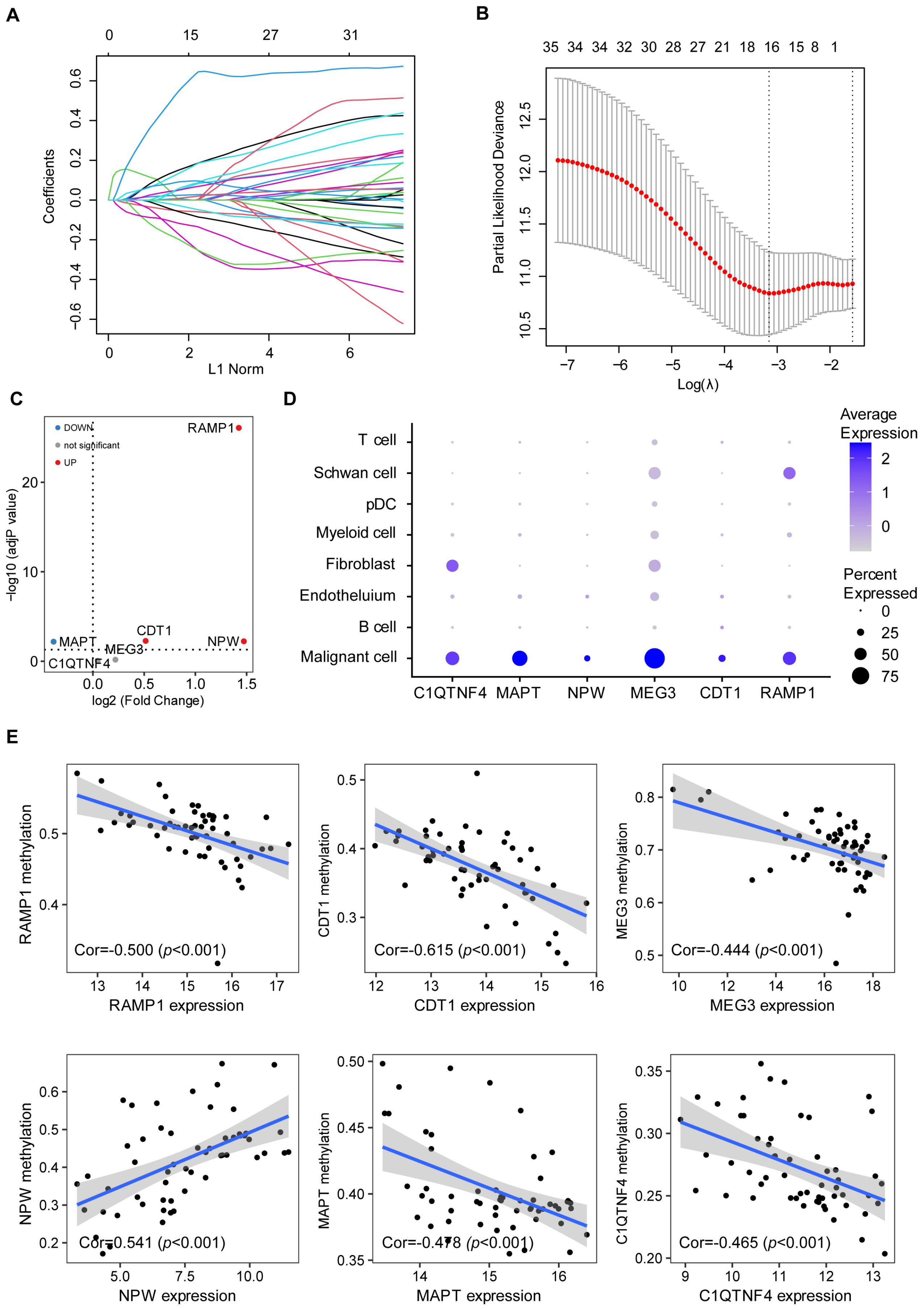

A
B
C
D
E
Coefficients
L1 Norm
Partial Likelihood Deviance
Log(λ)
−log10 (adjP value)
log2 (Fold Change)
DOWN
not significant
UP
RAMP1
MAPT
CDT1
NPW
MEG3
C1QTNF4
T cell
Schwan cell
pDC
Myeloid cell
Fibroblast
Endotheluium
B cell
Malignant cell
Average Expression
Percent Expressed
RAMP1 methylation
RAMP1 expression
Cor=-0.500 (p<0.001)
CDT1 methylation
CDT1 expression
Cor=-0.615 (p<0.001)
MEG3 methylation
MEG3 expression
Cor=-0.444 (p<0.001)
NPW methylation
NPW expression
Cor=0.541 (p<0.001)
MAPT methylation
MAPT expression
Cor=-0.478 (p<0.001)
C1QTNF4 methylation
C1QTNF4 expression
Cor=-0.465 (p<0.001)

Supplementary Figure 3. Prognostic model establishment for HRNB patients in SEQC cohort. **(A-B)** Identification of the optimal penalization coefficient lambda in the LASSO regression model. **(C)** groups. **(D)** Bubble plot showing the expression of six malignant cell marker genes in each cell type. Bubble intensity of the color indicated the average expression in a particular cell type, and bubble size represented the percentage of cells expressing the gene in that cell type. **(E)** The correlation between DNA methylation and mRNA expression of six malignant cell marker genes.

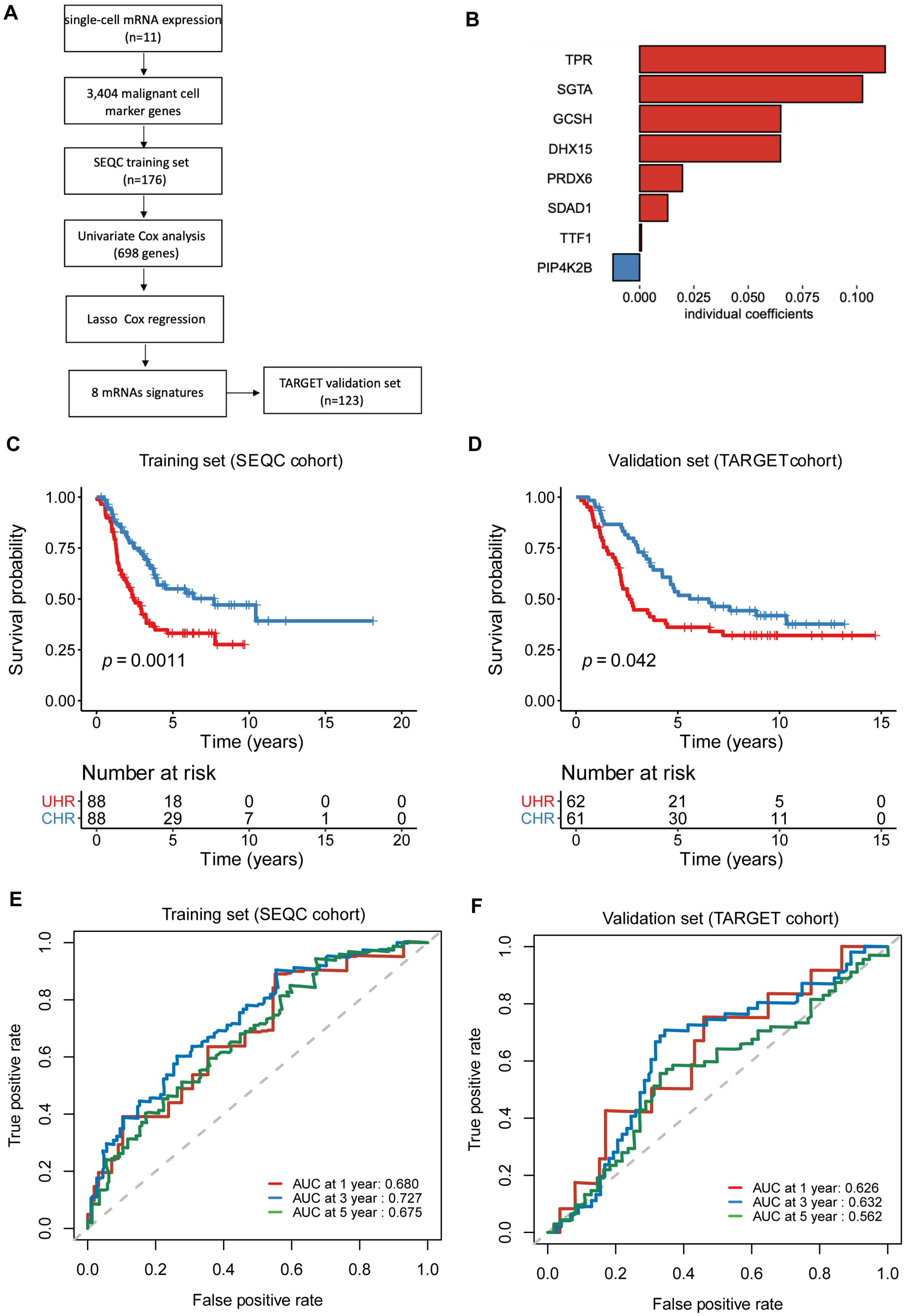
A
single-cell mRNA expression (n=11)
3,404 malignant cell marker genes
SEQC training set (n=176)
Univariate Cox analysis (698 genes)
Lasso Cox regression
8 mRNAs signatures
TARGET validation set (n=123)
B
TPR
SGTA
GCSH
DHX15
PRDX6
SDAD1
TTF1
PIP4K2B
0.000 0.025 0.050 0.075 0.100
individual coefficients
C
Training set (SEQC cohort)
Survival probability
p = 0.0011
Time (years)
Number at risk
UHR 88 18 0 0 0
CHR 88 29 7 1 0
D
Validation set (TARGET cohort)
Survival probability
p = 0.042
Time (years)
Number at risk
UHR 62 21 5 0
CHR 61 30 11 0
E
Training set (SEQC cohort)
True positive rate
False positive rate
AUC at 1 year: 0.680
AUC at 3 year : 0.727
AUC at 5 year : 0.675
F
Validation set (TARGET cohort)
True positive rate
False positive rate
AUC at 1 year: 0.626
AUC at 3 year : 0.632
AUC at 5 year : 0.562

Supplementary Figure 4. Model construction without METcor gene set. **(A)** Overall flowchart of model construction only without METcor gene set. **(B)** The coefficients of the identified eight signature genes. KM curves of OS between the UHR and CHR groups in the training set **(C)** and validation set **(D)**, respectively. ROC curves to predict the 1-, 3-, and 5-year OS in the training set **(E)** and validation set **(F)**, respectively.

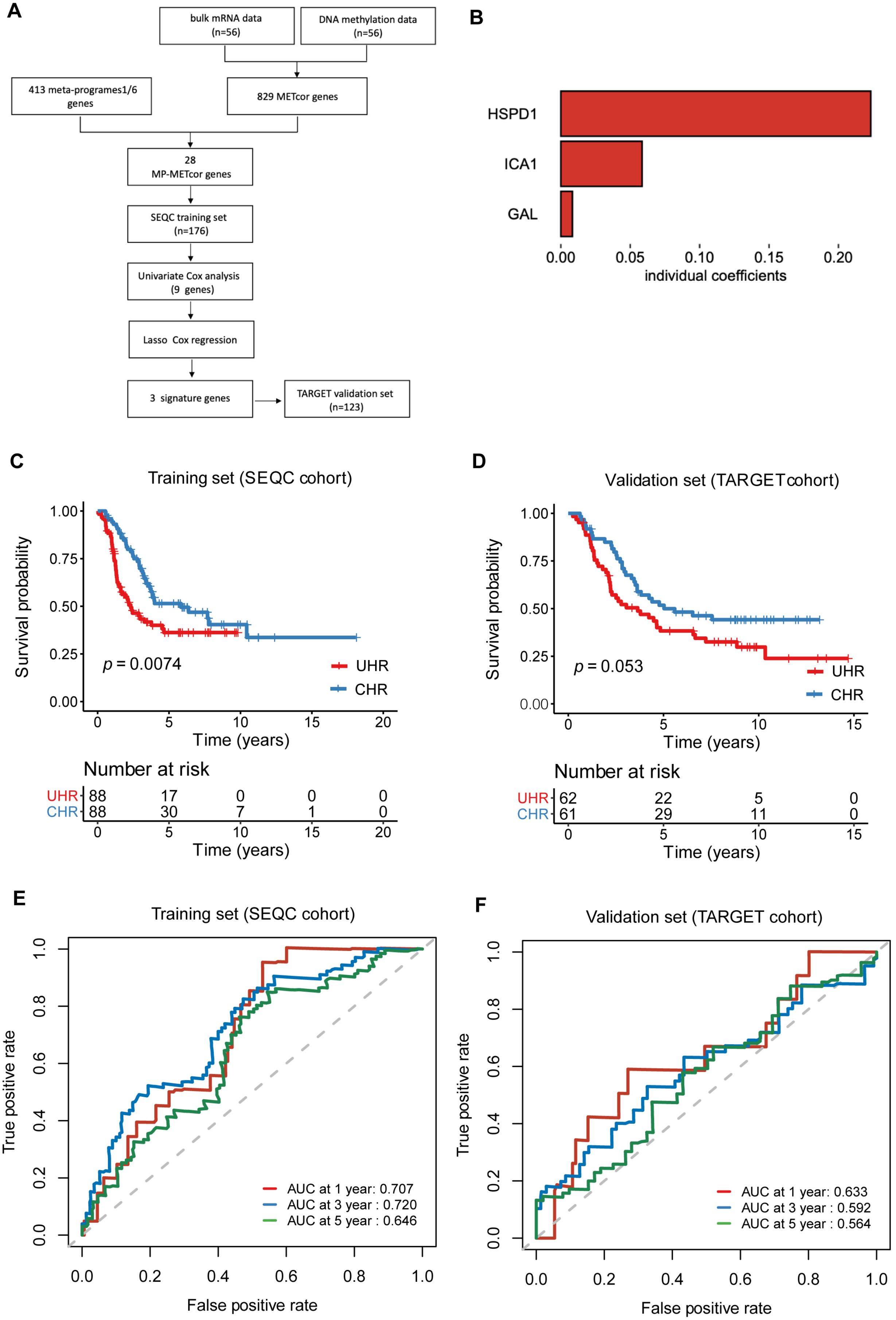
A
bulk mRNA data (n=56)
DNA methylation data (n=56)
413 meta-programes1/6 genes
829 METcor genes
28 MP-METcor genes
SEQC training set (n=176)
Univariate Cox analysis (9 genes)
Lasso Cox regression
3 signature genes
TARGET validation set (n=123)
B
HSPD1
ICA1
GAL
0.00
0.05
0.10
0.15
0.20
individual coefficients
C
Training set (SEQC cohort)
Survival probability
p = 0.0074
UHR
CHR
Time (years)
Number at risk
UHR 88 17 0 0 0
CHR 88 30 7 1 0
D
Validation set (TARGETcohort)
Survival probability
p = 0.053
UHR
CHR
Time (years)
Number at risk
UHR 62 22 5 0
CHR 61 29 11 0
E
Training set (SEQC cohort)
True positive rate
False positive rate
AUC at 1 year: 0.707
AUC at 3 year : 0.720
AUC at 5 year : 0.646
F
Validation set (TARGET cohort)
True positive rate
False positive rate
AUC at 1 year: 0.633
AUC at 3 year : 0.592
AUC at 5 year : 0.564

Supplementary Figure 5. Three-steps of model construction using metaprograms-METcor genes. **(A)** Overall flowchart of model construction using metaprograms-METcor genes. **(B)** The coefficients of the identified three signature genes. KM curves of OS between the UHR and CHR groups in the training set **(C)** and validation set **(D)**, respectively. ROC curves to predict the 1-, 3-, and 5-year OS in the training set **(E)** and validation set **(F)**, respectively. MP: metaprograms.

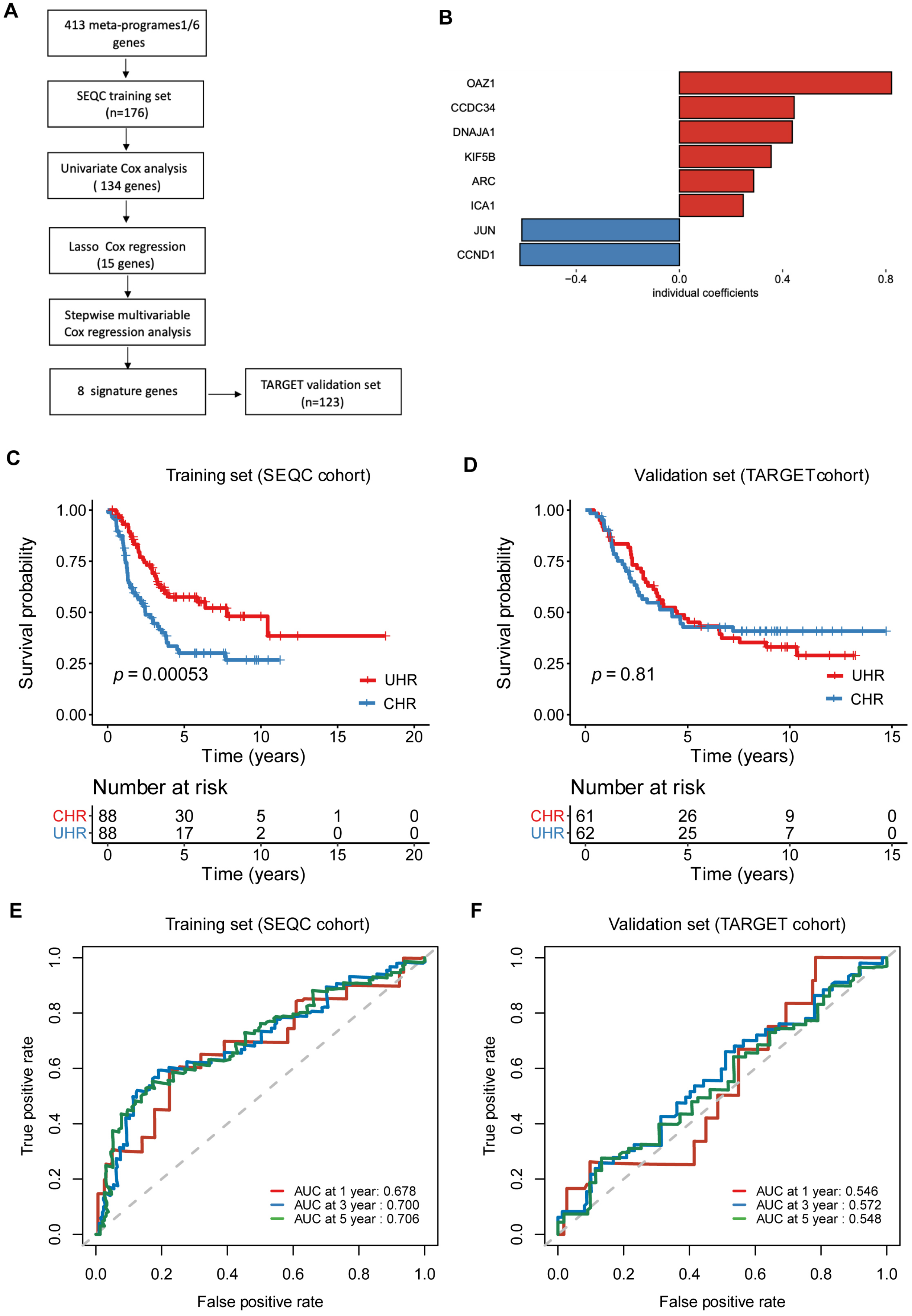
A
413 meta-programes1/6 genes
SEQC training set (n=176)
Univariate Cox analysis (134 genes)
Lasso Cox regression (15 genes)
Stepwise multivariable Cox regression analysis
8 signature genes
TARGET validation set (n=123)
B
OAZ1
CCDC34
DNAJA1
KIF5B
ARC
ICA1
JUN
CCND1
−0.4
0.0
0.4
0.8
individual coefficients
C
Training set (SEQC cohort)
Survival probability
1.00
0.75
0.50
0.25
0.00
p = 0.00053
UHR
CHR
0
5
10
15
20
Time (years)
Number at risk
CHR 88 30 5 1 0
UHR 88 17 2 0 0
0
5
10
15
20
Time (years)
D
Validation set (TARGETcohort)
Survival probability
1.00
0.75
0.50
0.25
0.00
p = 0.81
UHR
CHR
0
5
10
15
Time (years)
Number at risk
CHR 61 26 9 0
UHR 62 25 7 0
0
5
10
15
Time (years)
E
Training set (SEQC cohort)
True positive rate
0.0
0.2
0.4
0.6
0.8
1.0
AUC at 1 year: 0.678
AUC at 3 year : 0.700
AUC at 5 year : 0.706
0.0
0.2
0.4
0.6
0.8
1.0
False positive rate
F
Validation set (TARGET cohort)
True positive rate
0.0
0.2
0.4
0.6
0.8
1.0
AUC at 1 year: 0.546
AUC at 3 year : 0.572
AUC at 5 year : 0.548
0.0
0.2
0.4
0.6
0.8
1.0
False positive rate

Supplementary Figure 6. Three-steps of model construction using metaprograms genes without METcor gene set. **(A)** Overall flowchart of model construction using metaprograms genes without METcor gene set. **(B)** The coefficients of the identified eight signature genes. KM curves of OS between the UHR and CHR groups in the training set **(C)** and validation set **(D)**, respectively. ROC curves to predict the 1-, 3-, and 5-year OS in the training set **(E)** and validation set **(F)**, respectively.

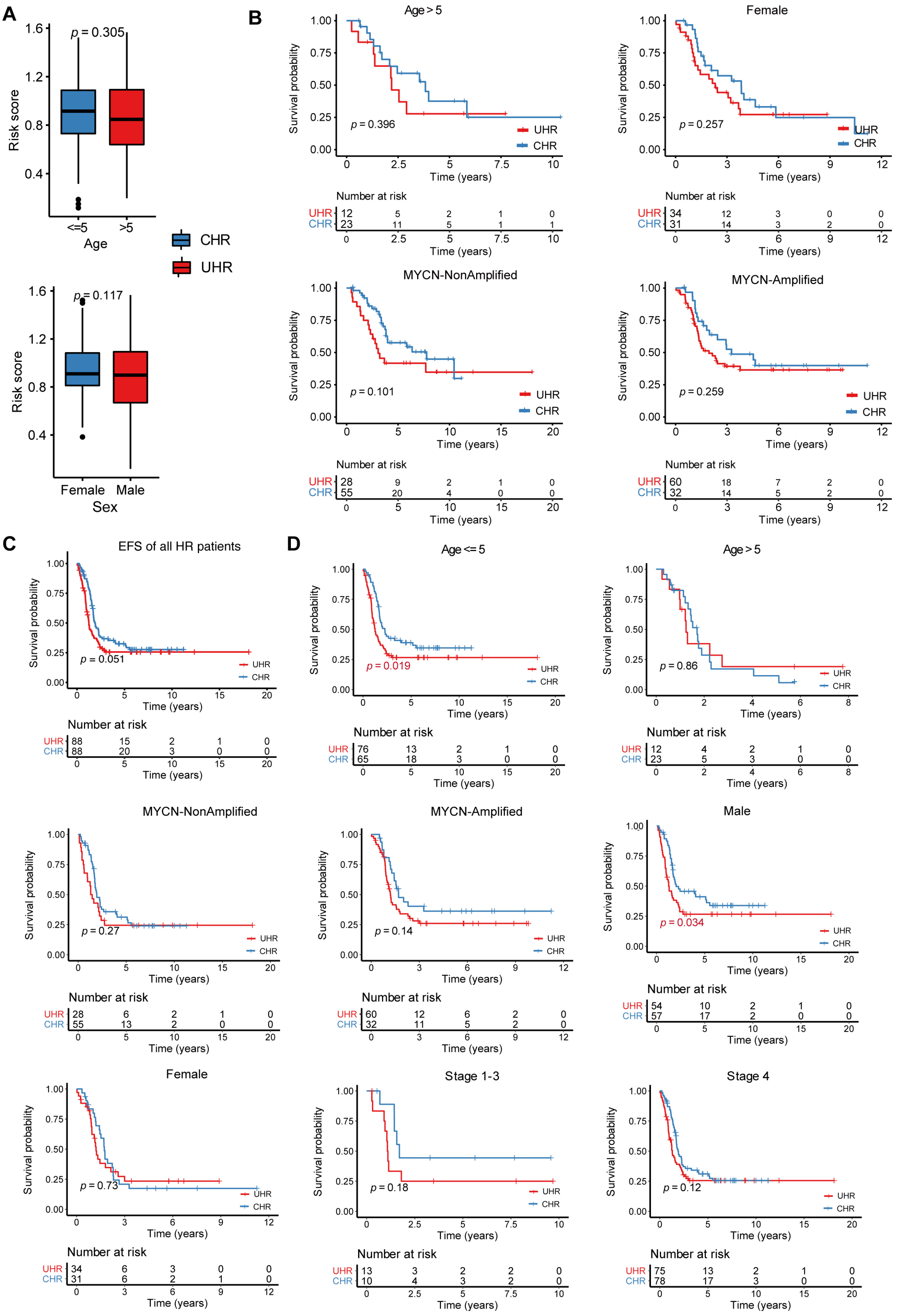
A
B
C
D
p = 0.305
Risk score
Age
<=5
>5
CHR
UHR
p = 0.117
Sex
Female
Male
Age > 5
p = 0.396
Female
p = 0.257
MYCN-NonAmplified
p = 0.101
MYCN-Amplified
p = 0.259
Survival probability
Time (years)
Number at risk
EFS of all HR patients
p = 0.051
Age <= 5
p = 0.019
Age > 5
p = 0.86
MYCN-NonAmplified
p = 0.27
MYCN-Amplified
p = 0.14
Male
p = 0.034
Female
p = 0.73
Stage 1-3
p = 0.18
Stage 4
p = 0.12

Supplementary Figure 7. The correlation of MMGS risk score with clinical features in the SEQC cohort. **(A)** The distribution of MMGS risk scores in different age and sex subgroups. **(B)** The difference in OS between UHR and CHR groups in patients with different clinical features. The difference in EFS between UHR and CHR groups in **(C)** all HR patients or **(D)** patients with different clinical features. EFS: event-free survival.

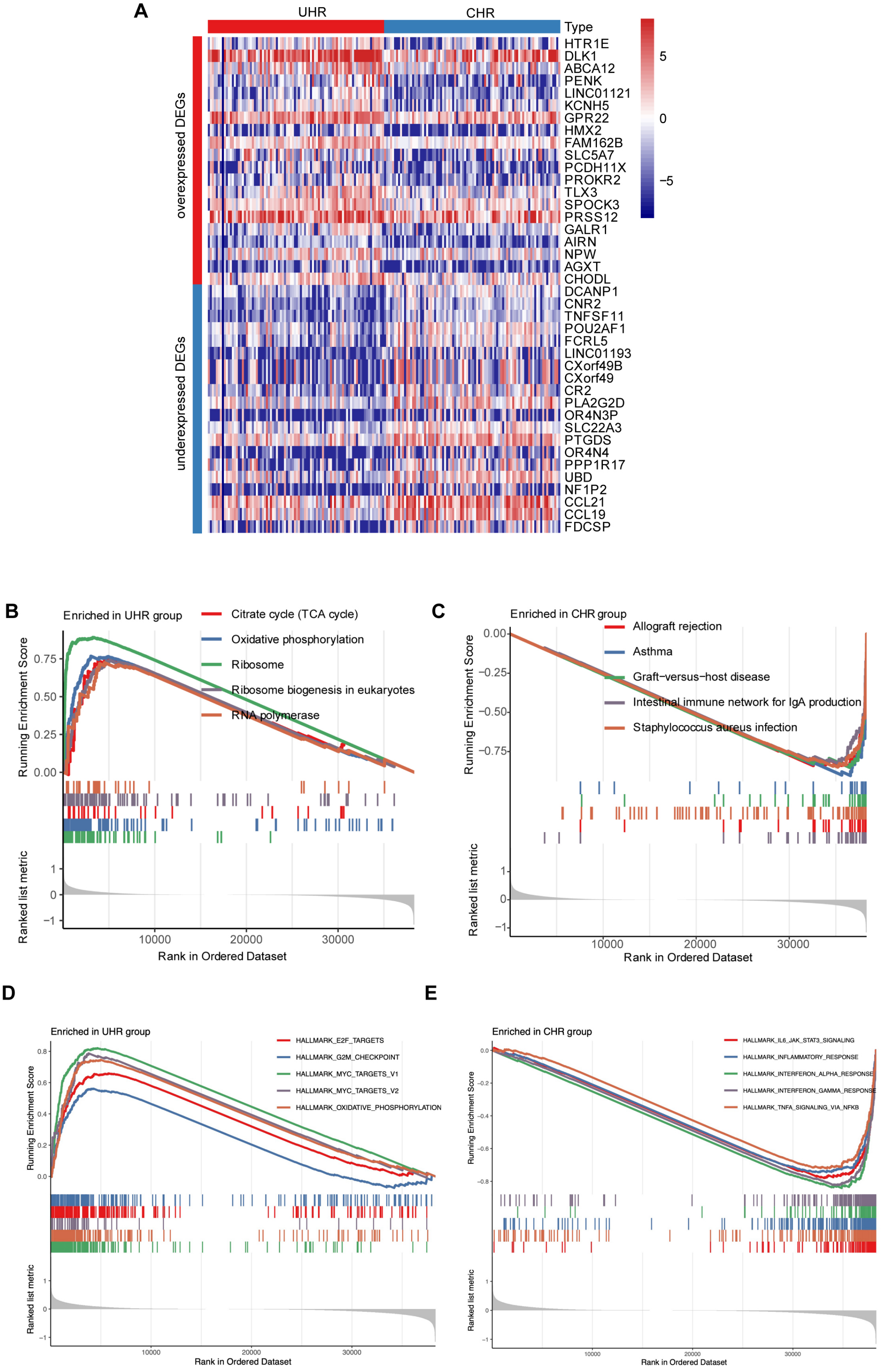
A
UHR
CHR
Type
overexpressed DEGs
underexpressed DEGs
HTR1E
DLK1
ABCA12
PENK
LINC01121
KCNH5
GPR22
HMX2
FAM162B
SLC5A7
PCDH11X
PROKR2
TLX3
SPOCK3
PRSS12
GALR1
AIRN
NPW
AGXT
CHODL
DCANP1
CNR2
TNFSF11
POU2AF1
FCRL5
LINC01193
CXorf49B
CXorf49
CR2
PLA2G2D
OR4N3P
SLC22A3
PTGDS
OR4N4
PPP1R17
UBD
NF1P2
CCL21
CCL19
FDCSP
5
0
−5
B
Enriched in UHR group
Citrate cycle (TCA cycle)
Oxidative phosphorylation
Ribosome
Ribosome biogenesis in eukaryotes
RNA polymerase
Running Enrichment Score
Ranked list metric
Rank in Ordered Dataset
C
Enriched in CHR group
Allograft rejection
Asthma
Graft−versus−host disease
Intestinal immune network for IgA production
Staphylococcus aureus infection
Running Enrichment Score
Ranked list metric
Rank in Ordered Dataset
D
Enriched in UHR group
HALLMARK_E2F_TARGETS
HALLMARK_G2M_CHECKPOINT
HALLMARK_MYC_TARGETS_V1
HALLMARK_MYC_TARGETS_V2
HALLMARK_OXIDATIVE_PHOSPHORYLATION
Running Enrichment Score
Ranked list metric
Rank in Ordered Dataset
E
Enriched in CHR group
HALLMARK_IL6_JAK_STAT3_SIGNALING
HALLMARK_INFLAMMATORY_RESPONSE
HALLMARK_INTERFERON_ALPHA_RESPONSE
HALLMARK_INTERFERON_GAMMA_RESPONSE
HALLMARK_TNFA_SIGNALING_VIA_NFKB
Running Enrichment Score
Ranked list metric
Rank in Ordered Dataset

Supplementary Figure 8. Representative genes and pathways enriched in the UHR and CHR groups in the SEQC and TARGET cohort, respectively. **(A)** The expression profiles of the top 20 overexpressed and underexpressed DEGs in the UHR group in the SEQC cohort. The top five significantly enriched KEGG pathways in the UHR **(B)** and CHR **(C)** HRNB patients in the TARGET cohort. The top five significantly enriched hallmarks in the UHR **(D)** and CHR **(E)** HRNB patients in the TARGET cohort.

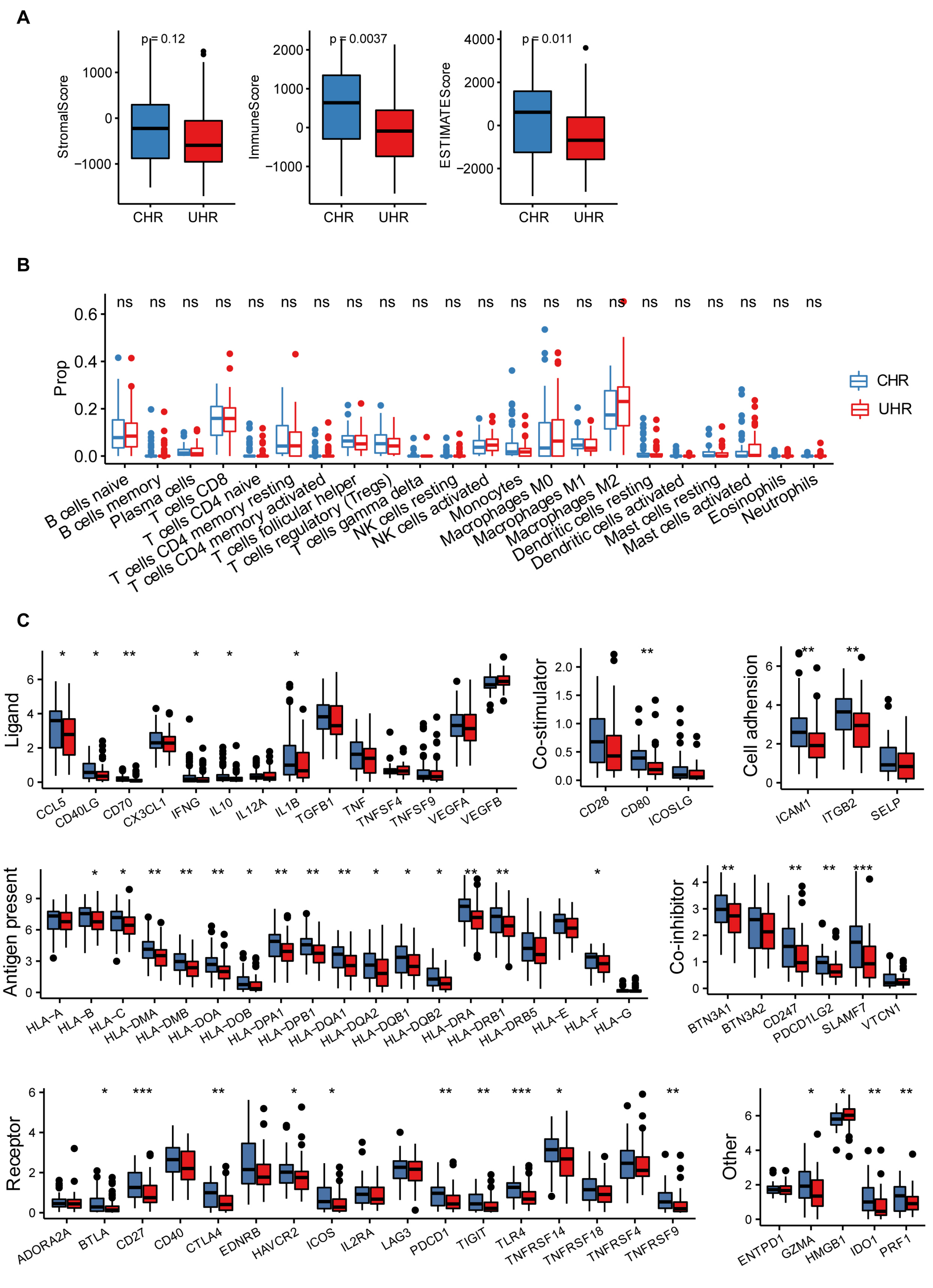

Supplementary Figure 9. The association between MMGS and the immune cell infiltration in the TARGET cohort. **(A)** Differences among stromal score, immune score, and ESTIMATE score between UHR and CHR groups. **(B)** The comparison of infiltration levels of 22 immune cell types between UHR and CHR groups. **(C)** Immune checkpoints expression between UHR and CHR groups. *$p < 0.05$; **$p < 0.01$; ***$p < 0.001$; ****$p < 0.0001$; ns: no significance.

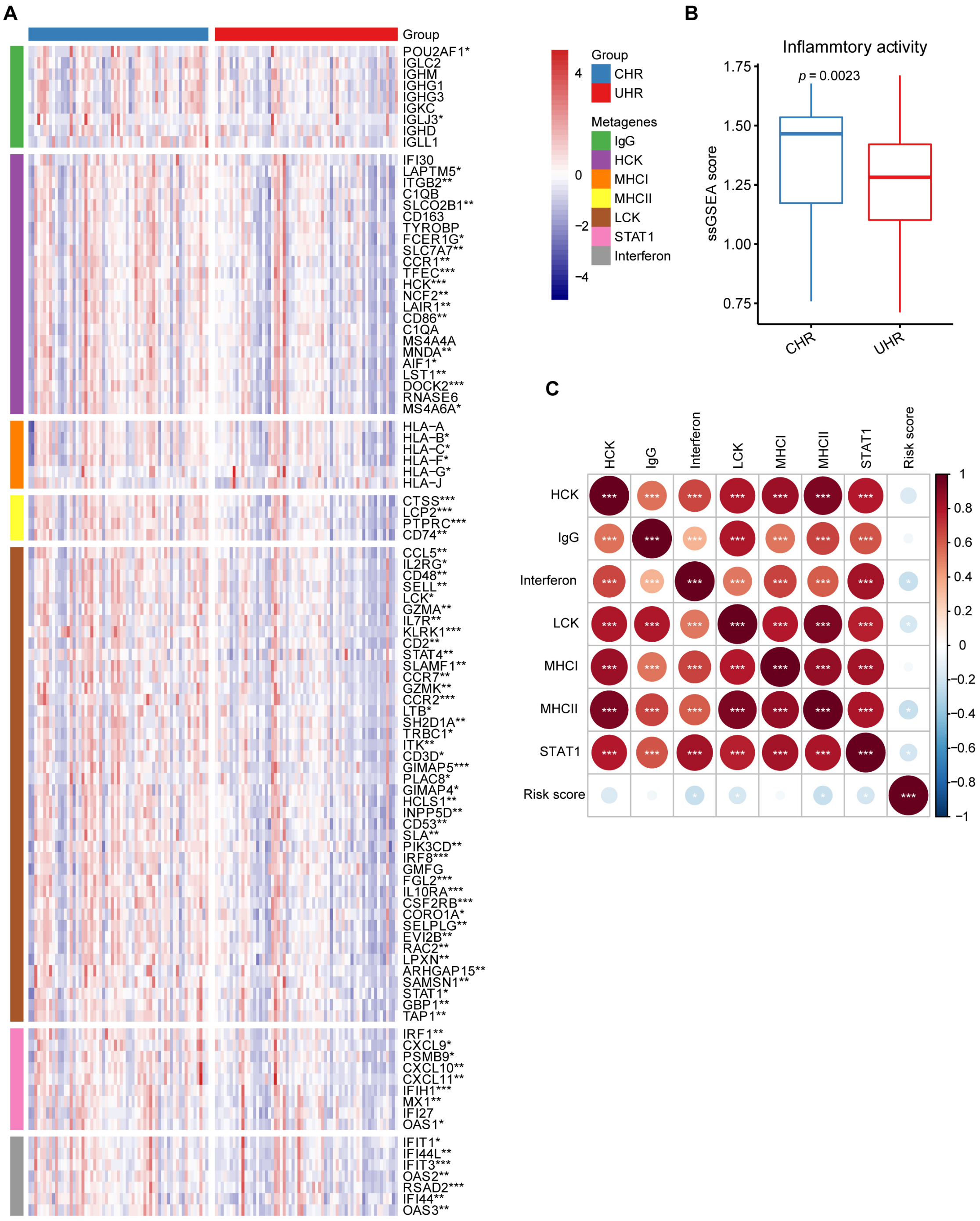

Supplementary Figure 10. Relationship between MMGS and inflammatory metagenes in the TARGET cohort. **(A)** Heatmap showing the expression characteristics of seven clusters of metagenes. **(B)** The ssGSEA score of inflammatory activity in MMGS risk groups. **(C)** The correlation between MMGS risk scores and metagenes. *$p < 0.05$; **$p < 0.01$; ***$p < 0.001$.

A **Training set (SEQC cohort)**

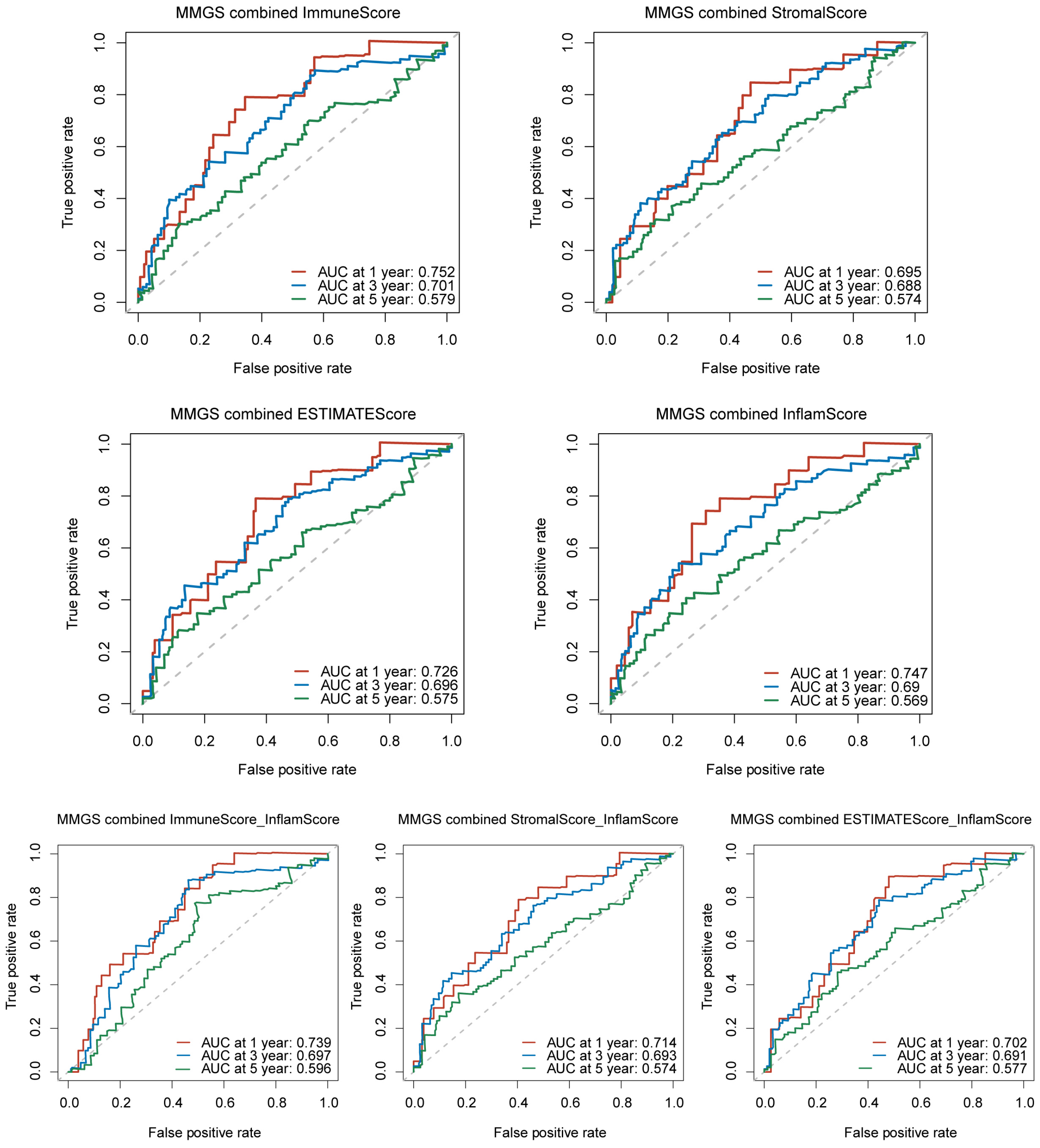

B

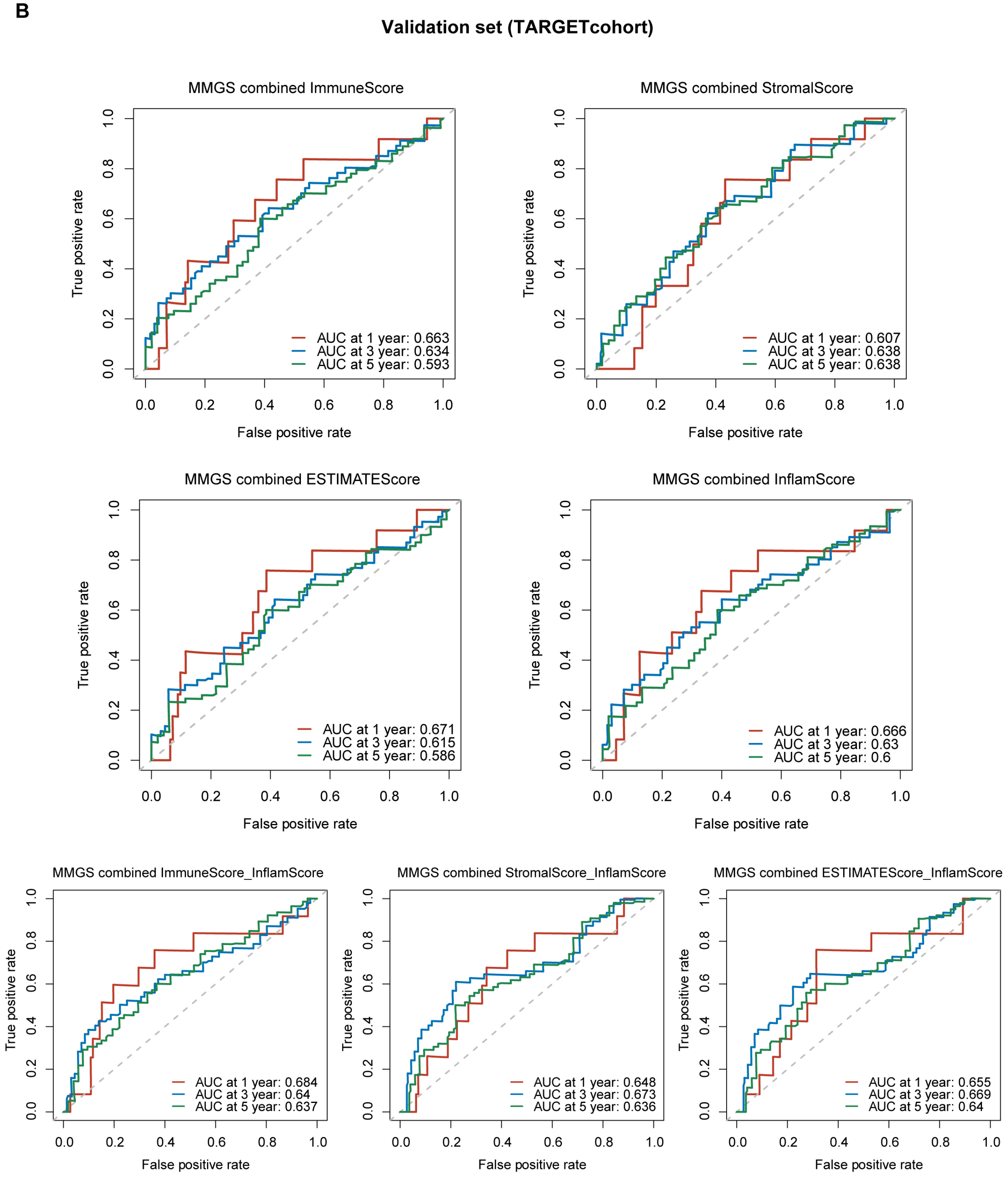

Supplementary Figure 11. ROC curves of MMGS combined immune and inflammatory features to predict the 1-, 3-, and 5-year OS in the training set **(A)** and validation set **(B)**, respectively.

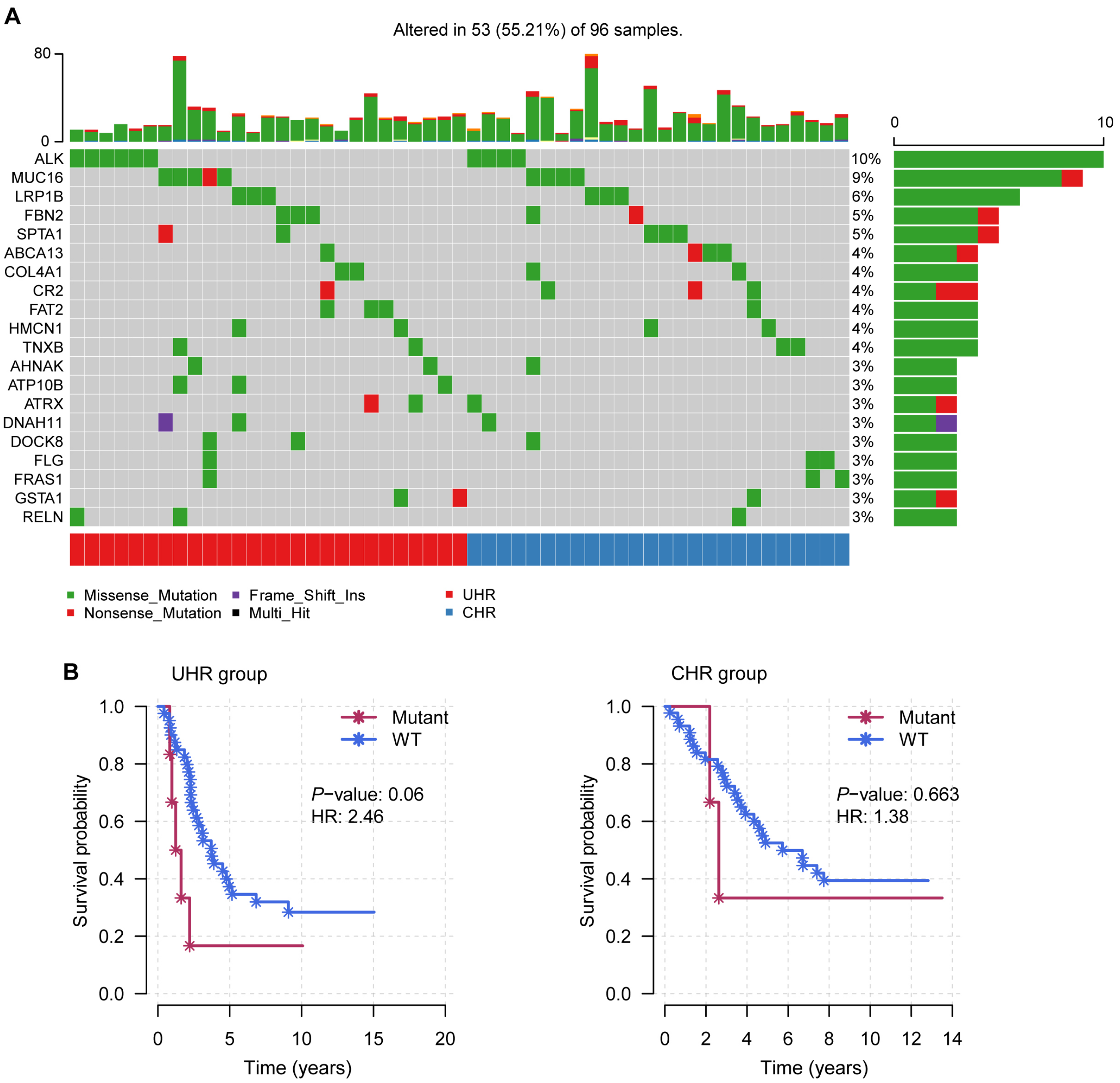

Supplementary Figure 12. Mutation landscape of MMGS risk groups from TARGET cohort. **(A)** The oncoPrint summarized the gene mutation landscape in the UHR and CHR groups. **(B)** KM curves of OS between patients with and without ALK mutation in the UHR and CHR group, respectively.
